# On the Selection of Transmission Range in Underwater Acoustic Sensor Networks

**DOI:** 10.3390/s120404715

**Published:** 2012-04-11

**Authors:** Mingsheng Gao, Chuan Heng Foh, Jianfei Cai

**Affiliations:** School of Computer Engineering, Nanyang Technological University, 639798, Singapore; E-Mails: mingsh.gao@gmail.com (M.G.); asjfcai@ntu.edu.sg (J.C.)

**Keywords:** underwater acoustic sensor networks, transmission range selection, energy efficiency, connectivity, random networks

## Abstract

Transmission range plays an important role in the deployment of a practical underwater acoustic sensor network (UWSN), where sensor nodes equipping with only basic functions are deployed at random locations with no particular geometrical arrangements. The selection of the transmission range directly influences the energy efficiency and the network connectivity of such a random network. In this paper, we seek analytical modeling to investigate the tradeoff between the energy efficiency and the network connectivity through the selection of the transmission range. Our formulation offers a design guideline for energy-efficient packet transmission operation given a certain network connectivity requirement.

## 1. Introduction

Typically, underwater acoustic sensor networks (UWSNs) consist of sensors that are deployed to perform collaborative monitoring tasks over a given region, such as oceanographic data collection, marine pollution monitoring, offshore exploration and disaster prevention and tactical surveillance [1–3]. UWSNs that require to monitor a large geographical area are often configured to operate in a multi-hop transmission mode. In other words, sensor nodes typically rely on neighboring sensor nodes to relay their transmissions to the default destination which is commonly called a sink for collecting sensor data.

Sensor nodes are prone to failures due to fouling and corrosion in the underwater environment. They are battery powered, which implies a limited operational lifetime. Due to the deployment remoteness of UWSNs, replacing faulty or flat sensor nodes incurs high cost. Thus, the deployment of UWSNs plays an important role in the *function, efficiency* and *reliability* of UWSNs, where (i) the function of UWSNs is related to the fulfillment of timely collection of sensor data, and sensing coverage and network connectivity are two common focuses; (ii) the efficiency of UWSNs is related to the energy consumption for the collection of a unit sensor data and for the operational maintenance of the UWSNs; and (iii) the reliability of UWSNs is related to the maintenance of the proper functions of UWSNs when some sensor nodes fail.

There are broadly two strategies in sensor node deployments. If a certain precision of location can be achieved in sensor node deployment, a precise planning of sensor node location can be sought to ensure full functions of complete sensing coverage and network connectivity based on a certain geometrical arrangement with the least number of sensor nodes [4–7]. The operational efficiency of such UWSNs can also be designed given the knowledge of sensor node geometrical arrangement. However, deploying sensor nodes precisely to their designated locations and maintaining their locations during the operation are often difficult underwater due to constant appearance of current in the environment. Moreover, achieving minimum redundancy of sensor nodes may not be desirable as it introduces low error resilience of operation in UWSNs when sensor nodes fail.

In this paper, we consider an UWSN with random sensor node deployment. As opposed to the high-precision deployment of sensor nodes, here sensor nodes are deployed at random locations with no particular geometrical arrangements, which forms a random network. As a result, the function and the efficiency of such UWSN can no longer be guaranteed. Full sensing coverage and network-wide connectivity may not be reached, and operation may not be optimized for energy efficiency

In the aspect of energy efficiency, one key influencing factor is the transmission power of each sensor node. Intuitively, when a higher transmission power is used in a packet transmission, the transmission can reach a longer distance, hence a fewer number of transmission relays is involved in delivering a packet to the sink. However, this fewer involvement in transmission relays is achieved at the expense of high energy consumption per transmission. Additionally, a larger transmission radius also introduces interference which may eventually translate into a higher overhead for each successful packet transmission. On the other hand, when a lower transmission power is used in a packet transmission, less energy is used for each packet transmission or relay. However, a higher number of transmission relays is required, which may result in a higher energy consumption for an end-to-end packet transmission. Thus, there exists an optimum transmission range that maximizes the energy efficiency or minimizes the energy consumption.

On the other hand, in the aspect of function, with the randomness in sensor node locations, the full coverage of sensing and communication may not be fulfilled. The effectiveness of sensor data collection is dictated by the network connectivity from a sensor to the sink. A particular transmission range setting leads to a certain probability of network connectivity where a longer range gives a higher probability of full network connectivity.

In [8], based on a simulation study, for an underwater environment, Porto and Stojanovic illustrated that the optimal transmission power is found to be the one that corresponds to *minimal connectivity*. In other words, a transmission power from a sensor node just enough to reach its nearest neighbor in the direction towards the final destination gives the optimal use of energy. Their work suggests that in a random network, each node determines its minimum connectivity and then tunes its transmission power accordingly during the operation. By operating at the minimum connectivity, the minimum overall power consumption can be achieved while still maintaining the network-wide connectivity. However, their conclusion is only valid for an ideal situation, where there is no overhead for each transmission and reception. In practice, there are always a minimum transmission power requirement for a packet transmission and a receiving energy consumption. Moreover, the suggested dynamic adjustment of transmission range in [8] also introduces additional hardware that adds extra cost to the deployment.

As opposed to the study in [8], we consider a common setup of UWSNs where the sensor nodes equip with only basic functions and thus the minimum connectivity that requires distance information cannot be obtained. In this case, the transmission range of sensor nodes is predetermined, and a tradeoff between energy efficiency and network connectivity arises. Shorter transmission ranges may offer higher energy efficiency in packet transmissions but risk losing network connectivity. Conversely, longer transmission ranges may maintain network connectivity but reduce energy efficiency in packet transmissions. In this paper, we seek analytical modeling to investigate this tradeoff for an UWSN where sensor nodes are randomly deployed. We first present the relationships among the transmission range, the average energy consumption and the connectivity. We show that to achieve operation at the optimal transmission range with a targeted network connectivity, a certain network density is required. We further illustrate that the use of multiple sink setup can significantly reduce the need for high network density while maintaining the targeted network connectivity.

We would like to point out that there have been a number of studies focusing on the optimal transmission range in the literature for both terrestrial networks and underwater networks. Most of the studies primarily focus either on how to enhance throughput by adjusting transmission range [8] (or interchangeably transmission power [9]), or on the optimal deployment patterns [4] (including the optimal ratio of the transmission range to the sensing range [10], and the minimum number of sensors [7]) by which full coverage and full connectivity over a given region can be realized. They usually assume sensors can be manually placed anywhere, which is not the case, particularly in underwater environments. To the best of our knowledge, jointly considering the energy efficiency and the network connectivity for the selection of the transmission range in such a random network scenario has not been studied before. The major contribution of this paper lies in an analytical framework to model the relationships between the transmission range, the average energy consumption and the connectivity. Such an analytical framework is important in the sense that it provides a means for network designer to appropriately design the deployment of an UWSN for joint energy-efficiency and network-connectivity considerations.

The remainder of this paper is organized as follows. In Section 2, we describe our considered UWSN model, followed by the derivations of the energy efficiency and the network connectivity in Section 3. In Section 4, we present numerical and simulation results and illustrate the optimal transmission range and the tradeoff between energy efficiency and network connectivity Finally we conclude this paper in Section 5.

## 2. Network Model

### 2.1. Underwater Acoustic Sensor Networks

**Figure 1.**
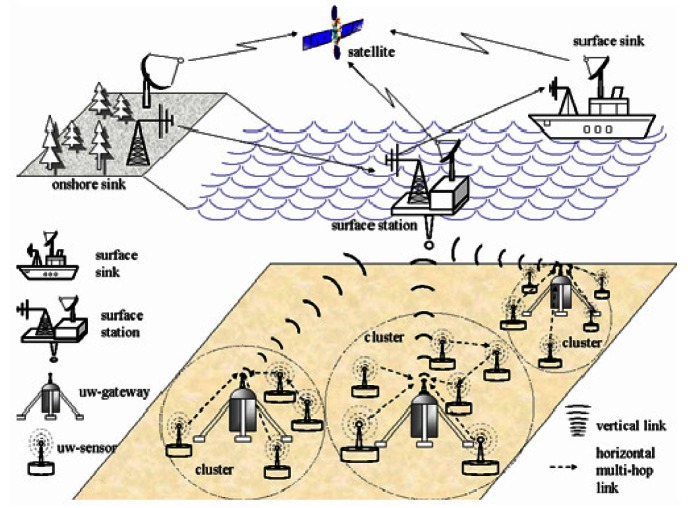
The network model for 2D UWSNs [1,11].

A reference architecture for two-dimensional underwater sensor networks is shown in Figure 1 (*cf.*, [1,11]), where deployed sensor nodes are anchored to the bottom of an ocean. Underwater sensors can be organized in a cluster-based architecture, and interconnected to one or more underwater gateways (U-GWs) through wireless acoustic links. A U-GW is equipped with a long-range vertical transceiver, which is used to relay data from the ocean bottom sensors to a surface station, and a horizontal transceiver, which is used to send commands and configuration data to the sensors as well as collecting monitored data. The surface station is equipped with an acoustic transceiver to handle multiple parallel communications with the U-GWs and a long-range radio or satellite transceiver to communicate with an onshore sink or a surface sink.

### 2.2. Basic Assumptions

To facilitate the analysis of an UWSN, we model it as follows. Suppose that the sink (denoted by *D*) is located at the center of a circle of radius *a*, where *a* is the largest possible distance between *D* and any sensor. Any sensor node (denoted by *S*), which intends to transmit a data packet to *D*, is assumed to be uniformly distributed over the entire circle. Due to the limited transmission energy, a packet from its originating source node to the sink may need to be sequentially routed by a certain number of intermediate nodes. For the sake of easy and practical deployment, we assume that all nodes, including the source node and the intermediate nodes, employ a common transmission range *r*. Consequently, direct transmission to the sink occurs only when the sensor node is within a distance of *r* from the sink. Any node within the transmission range of a node is called its neighbor. We assume that some routing protocol is employed so that each node can establish the shortest path to the sink.

As pointed out in [12], the selection of transmission range influences energy consumption and network connectivity. The question is how to quantitatively analyze the influence, which is described in detail in the next section.

## 3. Analysis of Energy Efficiency and Network Connectivity

In this section, we first describe the physical-layer underwater energy consumption model, which tells how large the energy consumption is in one transmission given a transmission range. Then, we analyze the average energy consumption w.r.t. an end-to-end packet transmission, *i.e.*, from a source node to the sink. After that, we provide the analysis on the connectivity for such a random network.

### 3.1. Underwater Energy Consumption Model

The attenuation or path loss that occurs in an underwater acoustic channel over a distance *l* for a signal of frequency *f* is given by

(1) $$A(l,f)=l^{k}\alpha {\left(f\right)}^{l}$$

where *k* is the spreading factor and *α*(*f*) is the absorption coefficient. The spreading factor *k* describes the geometry of propagation, and its commonly used values are *k* = 2 for spherical spreading, *k* = 1 for cylindrical spreading, and *k* = 1.5 for the so-called practical spreading. The counterpart of *k* in a radio channel is the path loss exponent whose value is usually between 2 and 4, where the former represents free-space line-of-sight propagation, and the latter represents two-ray ground-reflection model. The absorption coefficient *a*(*f*) in dB/km for *f* in kHz can be expressed as [13]:

(2) $$10\log\alpha (f)=0.11\frac{f^{2}}{1+f^{2}}+44\frac{f^{2}}{4100+f^{2}}+2.75\times 10^{-4}f^{2}+0.003$$

The above formula is generally valid for frequencies above a few hundred Hz. For lower frequencies, it is suggested to use the following formula:

(3) $$10\log\alpha (f)=0.002+0.11\frac{f^{2}}{1+f^{2}}+0.011f^{2}$$

The power consumption (denoted as *P_t_*(*l, f*)) for the single packet transmission with distance *l* and frequency *f* can be approximately expressed as [13]:

(4) $$P_{t}(l,f)=N(f)\;A(l,f)\;B(f)\text{SNR}$$

where *N*(*f*), *B*(*f*) and *SNR* are the power spectral density of the noise at frequency *f*, the usable bandwidth around the center frequency *f*, and the target signal-to-noise ratio at the receiver, respectively. The conversion from acoustic power *P_t_*(*l, f*) in dB re *μ*Pa to electrical power ${\text{P}}_{t}^{e}(\text{l})$ in Watt is given by [14]:

(5) $${\text{P}}_{t}^{e}(l)=P_{t}(l,f)\cdot 10^{-17.2}/\varphi$$

where 10^−17.2^ is the conversion factor and *φ* is the overall efficiency of the electric circuitry (power amplifier and transducer). Here *f* is omitted in ${\text{P}}_{t}^{e}(\text{l})$ for a fixed frequency

In practice, a certain non-zero minimum level of power is always radiated for a transmission regardless of how short the distance is [15]. Thus, the total power required for communicating over a distance *l* is modified as:

(6) $$P(l)=\max\left\{{\text{P}}_{t}^{e}\left(l\right),P_{\text{min}}\right\}+P_{\text{r}}$$

where *P_min_* is the minimum transmission power, *P_r_* is the fixed overhead for receiving data, and all of them are measured in Watt. Then, the total energy consumption for single transmission (denoted by *η*(*l*) in Jouel) is calculated as:

(7) $$\eta (l)=P(l)\times \frac{L_{d}}{R}$$

where *L_d_* is the packet size in bit and *R* is the transmission rate in bps.

### 3.2. Analysis of the Energy Efficiency

Here, we study the **one-hop energy-distance ratio,** which is defined as the ratio of the energy consumption for the one-hop transmission to the average **distance progress** of a packet during the one-hop transmission, where the **distance progress** refers to the difference between the before-hop distance (between the sender and the sink) and the after-hop distance (between the relay node and the sink) [15]. We consider that one-hop energy-distance ratio is able to represent the overall energy efficiency since any intermediate relay transmission can be viewed as a new one-hop transmission for the remaining route. The one-hop energy-distance ratio should be consistent with the overall energy-distance ratio for the entire route in a homogeneous environment, which will be later substantiated by simulations in Section 4. Note that the determination of the relationship between the transmission range and the energy efficiency is an extension of the works in [16,17] with different definitions, setups and derivations.

**Figure 2.**
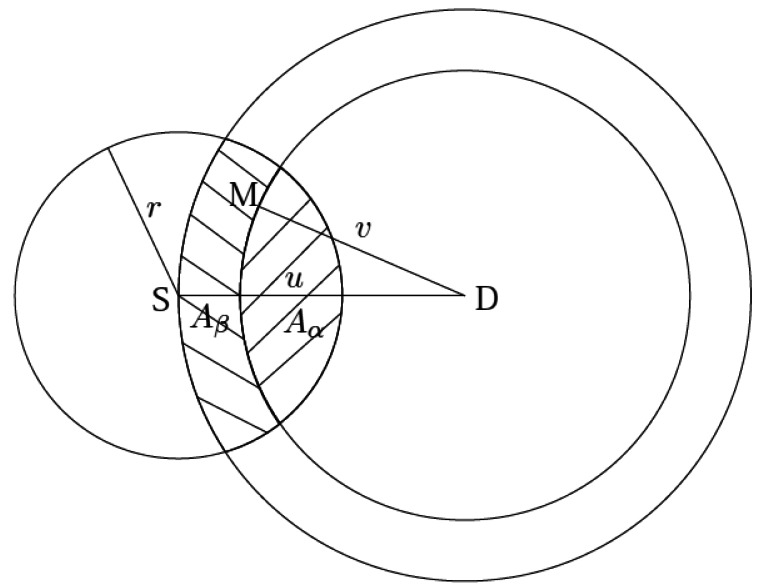
An illustration of the forwarding progress.

Let *u* be the distance between a sensor node *S* and the sink *D* as shown in Figure 2. The condition where *u* ≤ *r* gives a direct transmission from *S* to *D*, and the distance progress is *u*. For *u* > *r*, a neighboring node, say *M*, is required to relay packets between *S* and *D*, and the distance progress is equal to (*u − υ*) with *υ* being the distance between the one-hop router *M* and the sink *D*. In other words, a packet only travels (*u − υ*) distance towards *D*, and it has another *v* distance to travel.

Denote by 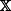 the random variable (r.v.) corresponding to the distance progress for a one-hop transmission, and denote 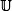 and 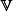 the r.v. for *u* and *υ*, respectively.

Like [17], we also assume that each node knows the locations of all its neighbors and the location of the destination node. We now define the **transmission strategy,** which can be described as follows: (i) The source node *S* directly transmits a packet to the destination node *D* if *D* is located within distance *r* from *S;* (ii) if the destination node *D* is outside the transmission range of the source node *S*, the packet is forwarded to the neighbor that is closer in distance to the destination node *D* than the source node *S*, and that is nearest to the source node *S* among all neighbors; and (iii) the source node S will not send out a packet when there does not exist any neighbor satisfying (ii), and will postpone the transmission until such a neighbor appears.

It should be pointed out that, the transmission strategy adopted in our study is **Nearest with Forward Progress** (NFP), while the transmission strategy adopted in [17] is **Most Forward with Fixed Radius** (MFR). This is due to the fact that (i) as pointed out in [8], a transmission power from a sensor node just enough to reach its nearest neighbor in the direction towards the final destination gives the optimal use of energy, and (ii) as shown in [16], NFP yields the highest one-hop throughput. Appropriate adjustments to the derivation based on [17] are made for our study. For completeness, in the following we provide the full derivation based on [17] with highlight of our adjustments.

Denote by 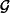 the event that there exists at least one relay node that is closer to the destination node than the source node if the destination node is outside the transmission range of the source node, but nearest to the source node among all neighbors, and denote by 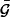 the complement of 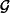 With the above denotations, 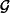 can be expressed as:

(8) $$\text{X}=\{\begin{array}{ll} \text{U}, & \text{if}\;\text{U}\leq r\\ \text{U}-\text{V}, & \text{if}\;\left(\text{U}>r\right)\cap G\\ 0, & \text{if}\;\left(\text{U}>r\right)\cap \bar{G} \end{array}$$

where at the third condition $\left(\text{U}>r\right)\cap \bar{G}$, there is no transmission since no route is established to the sink, and thus the progress is equal to zero.

As a result, the one-hop energy-distance ratio or the average energy consumption (denoted by *∈(r)* in J/m) is given by

(9) $$\begin{array}{ll} \in (r) & =\frac{\eta (r)}{E\left[\text{X}\right]}\\ & =\frac{\eta (r)}{E\left[\text{X}|\left(\text{U}\leq r\right)\cup \left(\left(\text{U}>r\right)\cap G\right)\right]} \end{array}$$

Note that (10)
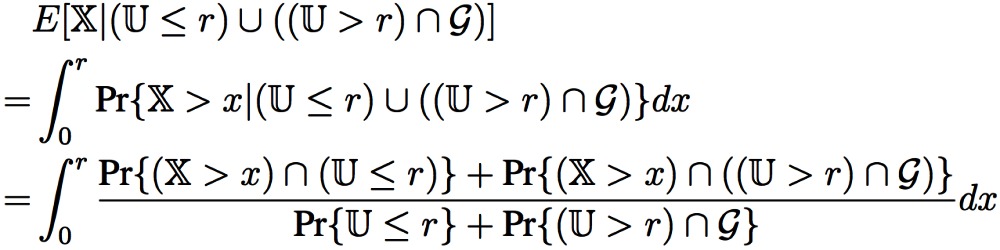
where there are four unknown terms: Pr{( 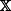 > *x*) ∩ ( 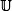 ≤ *r*)}, Pr{( 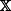 > *x*)∩ (( 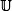 > r) ∩ 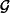}, and Pr{( 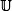 ≤ *r*)} and Pr{( 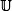 > *r*) ∩ 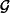}.

For the first unknown term, using Equation (8) gives Pr{( 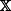 > *x*) ∩ ( 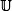 ≤ *r*)} = Pr{*x* < 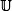 ≤ *r*} Then, from Figure 2, we see that

(11) $$\mathrm{Pr}\left\{x<\text{U}\leq r\right\}=\{\begin{array}{ll} \frac{r^{2}-x^{2}}{a^{2}}, & \text{if}x<r\\ 0, & \text{otherwise} \end{array}$$

where *a* is the largest possible distance between the sink *D* and any senor. Similarly, based on Figure 2, the third unknown term can be obtained by

(12) $$\mathrm{Pr}\left\{\text{U}\leq r\right\}=\frac{r^{2}}{a^{2}}$$

To compute the fourth unknown term, we denote by *A_s_* the area of the overlapping region between the circle centered at *S* with radius *r* and the circle centered at *D* with radius *u, i.e.*, the sum of the shaded regions *A_α_* and *A_β_* in Figure 2. Then

(13) $$\begin{array}{ll} \mathrm{Pr}\left\{\left(\text{U}>r\right)\cap G\right\} & =\int_{r}^{a}\mathrm{Pr}\left\{\text{at least one neighbor exist in}\;A_{s}\right\}f(u)du\\ & =\int_{r}^{a}\left(1-e^{-\rho A_{s}(u,r)}\right)\frac{2u}{a^{2}}du\\ & =1-\frac{r^{2}}{a^{2}}-\frac{2}{a^{2}}\int_{r}^{a}ue^{-\rho A_{s}(u,r)}du \end{array}$$

where *f*(*u*) is the probability density function (PDF) of 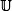, and differentiating Equation (12) gives $f(u)=\frac{2u}{a^{2}}$. Note that the second identity in Equation (13) comes from the assumption that the probability of having *n* nodes in an area of size *A* complies with a Poisson distribution, *i.e.*, (*ρA*)*^n^e^−ρA^/n*!, where *ρ* is the density parameter indicating the number of sensors per unit area [6]. The geometry of Figure 2 gives $A_{s}(u,r)=r^{2}\cos^{-1}\left(\frac{r}{2u}\right)+u^{2}\cos^{-1}\left(1-\frac{r}{2u^{2}}\right)-\frac{1}{2}r\sqrt{\left(2u+r)(2u-r\right)}$.

According to Equation (8) and Figure 2, the second unknown term can be determined by

(14) $$\begin{array}{ll} \mathrm{Pr}\{(X>x)\cap ((\text{U}>r)\cap G)\} & =\mathrm{Pr}\{(\text{U}-\text{V}>x)\cap (\text{U}>r)\cap G\}\\ & =\int_{r}^{a}\mathrm{Pr}\{(\text{V}<u-x)\cap G\}f(u)du\\ & =\int_{r}^{a}\mathrm{Pr}\{\text{there is no neighbor in }A_{\beta }\}f(u)du\\ & =\{\int_{r}^{a}\begin{array}{cc} (1-e^{-\rho (A_{s}(u,r)-A_{\alpha }(u-x,x,r))})\frac{2u}{a^{2}}du, & \text{if }x<r\\ 0, & \text{otherwise} \end{array} \end{array}$$

where *A_α_*(*υ, u, r*) is the overlapping region between the circle centered at *S* with radius *r* and the circle centered at *D* with radius *v*, as shown in Figure 2, and

$$A\alpha (\upsilon ,u,r)=r^{2}\cos^{-1}\left(\frac{r^{2}+u^{2}+\upsilon^{2}}{2ur}\right)+\upsilon^{2}\cos^{-1}\left(\frac{\upsilon^{2}+u^{2}-r^{2}}{2u\upsilon }\right)-\frac{1}{2}\sqrt{\left(u+\upsilon +r)(u+r-\upsilon )(u+\upsilon -r)(r+\upsilon -u\right)}$$

It is to note that the event {*there is no neighbor in A_β_*} in Equation (14) will be {*at least one neighbor in A_α_*} if the MFR strategy is adopted as in [17].

Substituting Equations (11), (12), (13) and (14) into Equation (10), then we obtain the result of Equation (10). Finally, we complete the calculation of Equation (9) by combing Equations (7) and (10).

### 3.3. The Network Connectivity

As aforementioned, minimizing the energy-distance ratio *∈*(*r*) in Equation (9) will lead to an optimal transmission range *r* that achieves minimal energy consumption. However, the optimal transmission range might cause some connectivity problem, since sensors are assumed to be placed uniformly and randomly in a fixed area. Thus, the selection of the transmission range needs to consider the connectivity requirement. In this section, we analyze the network connectivity given a network radius *a*, a node density *ρ* and a transmission range *r*. Here the network **connectivity** is defined as the probability that a sensor node can find at least one path to reach the sink (*i.e.*, node *D*).

Let *u_k_, υ_k_* and *x_k_, k* = 1, 2,…, be the distance between the *k^th^* forwarding node and sink *D*, the distance between its one-hop router and sink *D*, and the *k^th^* distance progress, respectively. Accordingly, denote r.v.s 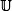
*_k_*, 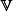
*_k_* and 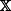
*_k_*, respectively, correspond to *u_k_, υ_k_* and *x_k_*. It is easy to see that *u_k+_*_1_ = *u_k_ − x_k_* for *k* =1, 2,….

Based on the definitions and Figure 2, we can derive the following conditional probability distribution function

(15) $$\begin{array}{ll} F\left(x_{k}|u_{k}\right) & ≐\mathrm{Pr}\left\{{\text{X}}_{k}\leq x_{k}|G^{k}\cap ({\text{U}}_{k}=u_{k})\right\}\\ & =\mathrm{Pr}\left\{u_{k}-{\text{V}}_{k}\leq x_{k}|G^{k}\cap \left({\text{U}}_{k}=u_{k}\right)\right\}\\ & =\mathrm{Pr}\left\{{\text{V}}_{k}\geq u_{k}-x_{k}|G^{k}\cap \left({\text{U}}_{k}=u_{k}\right)\right\}\\ & =e^{-\rho A_{\alpha }^{k}\left(u_{k}-x_{k},u_{k},r\right)}-e^{-\rho {\text{A}}_{s}^{k}\left(u_{k},r\right)} \end{array}$$

where ${\text{A}}_{s}^{k}$, $A_{\alpha }^{k}$ and *G^k^* are the counterparts in the *k^th^* forwarding of *A_s_, A_α_* and 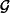 defined in previous subsection. Here we would like to stress that the triplet $\left({\text{A}}_{s}^{k},A_{\alpha }^{k},G^{k}\right)$ is indeed correlated to the triplet $\left(A_{s}^{k-1},A_{\alpha }^{k-1},G^{k-1}\right)$. They, however, can be assumed to be independent of each other because UWSNs are generally deployed in a sparse way.

Further, we define $g_{k}\left(x_{k}|u_{k}\right)=\frac{dF\left(x_{k}|u_{k}\right)}{dx_{k}}$. Let *P_k_*(*ρ,u*_1_) be the conditional probability that the source node *S* can be connected to the destination node *D* through *k* times forwarding with the initial distance between *S* and *D* being *u*_1_. By [18], assuming each forwarding is independent, *p_k_*(*ρ, u*_1_) can be approximately computed as:

(16) $$p_{k}\left(\rho ,u_{1}\right)={\int_{0}^{r}g_{1}\left(x_{1}|u_{1}\right)\int_{0}^{r}g}_{1}\left(x_{2}|u_{2}\right)\cdots \int_{0}^{r}gk\left(x_{k}|u_{k}\right)f\left(u_{k}\right)dx_{1}\cdots dx_{k}$$

where

(17) $$f\left(u_{k}\right)=\{\begin{array}{ll} 1, & \text{if}u_{k}\leq r\\ 0, & \text{otherwise} \end{array}$$

Thus, the connectivity of the network (denoted by *p_c_*) can be obtained as:

(18) $$p_{c}=\sum_{k}\int_{0}^{a}pk\left(\rho ,u_{1}\right)\frac{1}{a}du_{1}$$

Note that r.v. 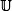 here is assumed to be uniformly distributed in the interval [0, *a*].

## 4. Performance Evaluation

We conduct simulation experiments to validate our analytical framework. The network coverage area is assumed to be a circle with radius ranging from 5,000 m to 15,000 m, and the sink is fixed at the center. The central frequency *f*, the frequency bandwidth *B*(*f*) and the target signal-to-noise ratio *SNR* in Equation (4) are set to 20 kHz, 2 kHz and 20 dB, respectively. The minimum transmitter power *P_min_* and *P_r_* in Equation (6) are set to 8 W [19] and 1 W, respectively. We choose the packet size *L_d_* to be 1,024-bit, and the transmission rate *R* to be 2 kbps. The conversion factor *φ* in Equation (5) is set to 0.25. The node density *ρ* is measured in the number of sensors per square meter (*m*^2^). All the results obtained are the average over 500 randomly selected topologies.

### 4.1. Results on The Energy Efficiency

**Figure 3.**
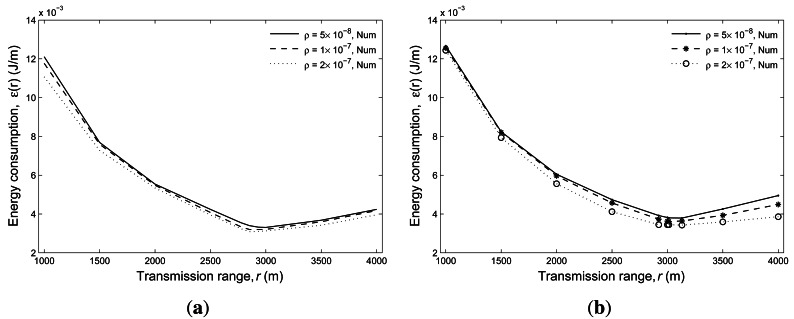
The numerical results of the average energy consumption *versus* the transmission range. (**a**) *a* = 5, 000 m; (**b**) *a* = 10, 000 m.

Figure 3 shows the numerical results of the energy consumption *versus* the transmission range with different node density *ρ* and different covering radius *a*. From all these results, it can be seen that with the increase of the transmission range *r*, the energy consumption decreases first but later increases after reaching a certain point. Such a pattern can be explained as follows. As the transmission range *r* increases, the probability of finding relay nodes closer to the sink would be higher, leading to a larger distance progress. Note, however, that the minimum transmission power is fixed at 8 W for small values of *r*. As a result, a larger *r* (<3, 000 m) renders a lower energy consumption. On the other hand, as can be seen from Equations (1) and (4), the transmission power becomes very large for large values of *r*, which increases exponentially with the increase of *r*. Despite the fact that a larger transmission range would induce a larger distance progress, the energy consumption per unit distance (e.g., per meter) still increases as the transmission range *r* becomes large. That is why we see a minimum point in the curve *∈*(*r*) which represents an optimal transmission range in energy consumption.

From Figure 3, we can also see that a larger *ρ* introduces a lower *∈*(*r*). For example, for Figure 3(a), when *r* = 3000 m, the values of *∈*(*r*) with *ρ* = 5 × 10^−8^, 1 × 10^−7^, 2 × 10^−7^ are 0.00331, 0.00321 and 0.00313, respectively. This can be attributed to the fact that a larger node density makes a larger one-hop progress under the same transmission range, rendering a lower energy consumption. The same conclusions still hold for Figure 3(b), where *a* = 10, 000 m.

**Figure 4.**
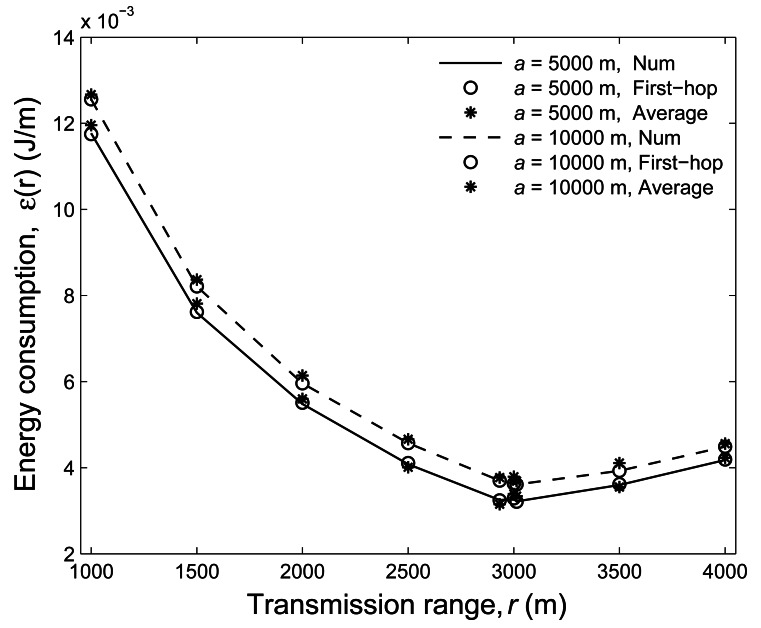
Comparisons of the numerical and simulation results of the average energy consumption under different transmission ranges with *ρ* = 10^−7^.

To validate our analytical result for the energy efficiency, in Figure 4, we compare the numerical and simulation results for *a* = 5, 000 m and 10, 000 m with *ρ* = 10^−7^. Clearly, both results reach a good agreement indicating the accuracy of our analytical approach. Similar to that of Figure 3, the relationship between transmission range and energy consumption still holds. Note that we also show the simulation results of the first-hop transmission, which demonstrates that the result of the one-hop transmission is consistent with that of the overall transmission.

**Figure 5.**
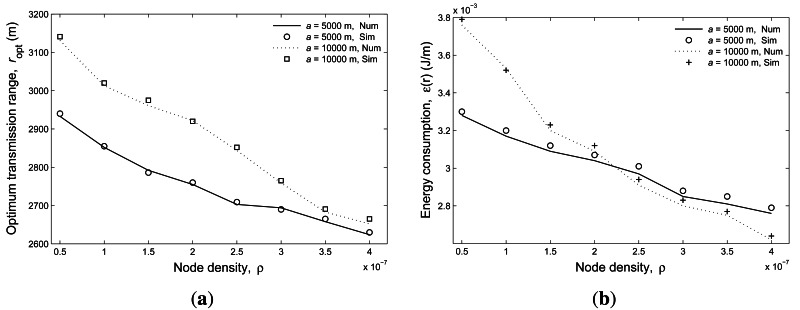
The optimal transmission range (**a**) and the corresponding energy consumption (**b**) under different node density values.

Figure 5(a) shows the optimal transmission range (*r_opt_*) *versus* the node density for both *a* = 5, 000 m and 10,000 m. Again, the close match between the simulation and analytic results is demonstrated. In addition, two observations can be made from this figure. First, the optimal transmission range decreases as the increasing of *ρ*. This is due to the increase in relative one-hop progress with respect to the transmission range. A smaller transmission range achieves better energy efficiency when *ρ* is larger. Second, a larger network radius *a* introduces a larger optimal transmission range in the case of the same *ρ*. This is because larger area corresponds to larger number of hops, which introduces more overhead. Figure 5(b) gives the corresponding average energy consumption results with the optimal transmission ranges.

### 4.2. Results on the Network Connectivity

**Figure 6.**
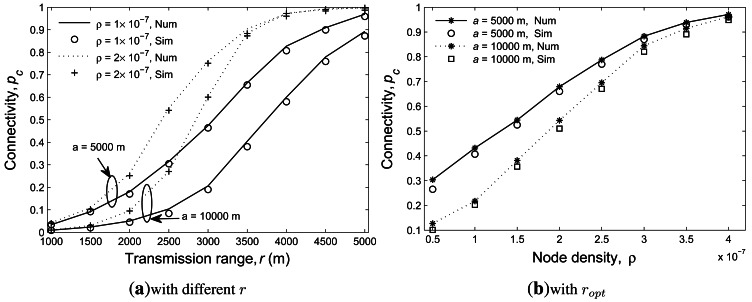
The results of the network connectivity with different transmission ranges (**a**) and with optimal transmission ranges (**b**).

Figure 6(a) shows the results of the connectivity probability *p_c_* under different transmission range *r* varying from 1,000 m to 5,000 m with *ρ* being either 1 × 10^−7^ or 2 × 10^−7^. Again, it demonstrates that the analytic and simulation results match closely. Moreover, it can be seen that a larger *ρ* results in a greater connectivity. This is intuitive because a larger *ρ* will increase the probability of having one-hop neighbors of any sensor node, thus rending a higher connectivity.

Figure 6(b) shows the connectivity with the optimal transmission range under different node density values. It can be seen that for a certain *a* and *ρ*, the optimal transmission range that achieves minimum average energy consumption might not lead to an satisfactory connectivity. For example, when *a* = 5, 000 m and *ρ* = 1 × 10^−7^, the optimum transmission range is 2,852 m, resulting an average energy consumption of 0.00317 J/m but a connectivity of 0.4317, which does not meet the practical connectivity requirement for UWSNs. Thus, the selection of the transmission range needs to take into consideration the tradeoff between the energy consumption and the connectivity. In particular, a targeted network connectivity requirement can be fulfilled by increasing either the node density or the transmission range, each of which incurs a cost. Increasing node density introduces additional hardware cost while increasing transmission range causes higher operational energy and transmission interference. Our formulation enables network designers to determine the tradeoff and thus derive an adequate setup to meet the requirements.

### 4.3. Determination of the Optimal *r* for Practical Applications

So far, we have investigated two relationships: one is between the transmission range and energy consumption, and the other is between the transmission range and the network connectivity. Given the network size, the node density and the threshold of the network connectivity, we may determine the optimal transmission range for sensor nodes to operate. In other words, we may adjust the transmission power of sensor nodes such that the energy consumption in transmissions is optimally set.

It is easy to see that, for a given transmission range, the energy efficiency and the network connectivity can be calculated using Equations (9) and (18), respectively. It is important to point out that the network connectivity *p_c_* is a monotonically increasing function with regard to the transmission range while the energy efficiency *∈*(*r*) is not. Motivated by this, here we propose a simple strategy to find the optimal transmission range.

First, for a particular threshold of the network connectivity, we can find the lowest transmission range, say *r*_1_, by using Equation (18) such that it satisfies the threshold of the network connectivity. Next, we determine the optimal transmission range, say *r*_2_, based on Equation (9). Finally, to ensure both the network connectivity requirement and energy efficiency, we simply take the largest value between *r*_1_ and *r*_2_ as the transmission range for operation.

### 4.4. The Multiple Sink Setup

In our earlier discussions, we suggested increasing either node density or transmission range to achieve a certain network connectivity requirement. In this subsection, we demonstrate the employment of a multiple sink setup as an alternative solution to meet the requirement.

**Figure 7.**
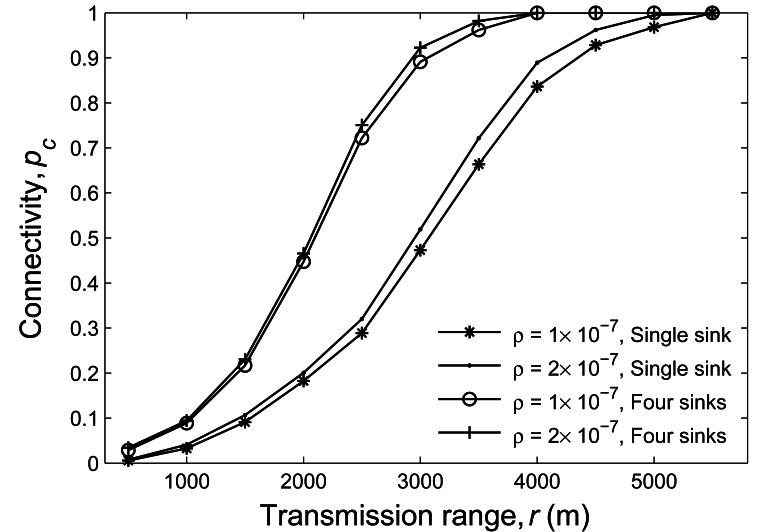
The simulation results of the network connectivity *versus* transmission range for single and multiple sink setups.

Assuming that a number of sensors are randomly deployed over a square area with the side length of 5,000 m, we consider two scenarios here: one is the scenario where there is only one sink located at the center of the square area, and the other is that there are four sinks individually placed at four different vertexes of the square. The node density *ρ* is set to either 1 × 10^−7^ or 2 × 10^−7^, and the transmission range varies from 1,000 m and 5,000 m. We evaluate the impact of multiple sinks on the connectivity, as shown in Figure 7. It is clear that the scenario with four sinks achieves higher connectivity than that with single sink.

## 5. Conclusions

In this paper, we developed an analytical framework which describes the relationship between the transmission range and the energy efficiency as well as the relationship between the transmission range and the connectivity in an UWSN scenario. We illustrated that the selection of the transmission range needs to consider the tradeoff between the energy efficiency and the connectivity. Meeting a certain level of network connectivity incurs either cost for additional node deployment or energy due to operational deviation from the optimal transmission range. Our analytical framework provides a means for network designers to plan the deployment of an UWSN. We further illustrated that employing multiple sinks helps to meet the connectivity requirement in a more cost-effective way.

Although we consider the underwater environment in this paper, the developed analytical framework is general and can be applied to other random network scenarios. In the future, we shall extend our investigation by considering a medium access control (MAC) protocol such as [20,21] in the computation. This allows us to capture the effect of transmission interference (e.g., due to transmission collisions) and energy overhead (e.g., due to retransmissions) in our formulations.
